# Changes in anthropometric and fitness profile of Italian regional academy rugby union players

**DOI:** 10.5114/biolsport.2022.106384

**Published:** 2021-07-28

**Authors:** Alexandru Nicolae Ungureanu, Luca Beratto, Federico Abate Daga, Gennaro Boccia, Corrado Lupo, Paolo Riccado Brustio

**Affiliations:** 1 NeuroMuscularFunction Research Group; School of Exercise & Sport Sciences, SUISM; University of Turin, Turin, Italy; 2 Department of Medical Sciences; University of Turin, Turin, Italy; 3 School of Exercise & Sport Sciences, SUISM; University of Turin, Turin, Italy; 4 Department of Clinical and Biological Sciences; University of Turin, Turin, Italy; 5 Department of Neuroscience, Biomedicine and Movement; University of Verona, Verona, Italy

**Keywords:** Youth rugby union, Anthropometry Player profiling, Fitness testing, Long term development

## Abstract

In rugby union, physical characteristics may partially contribute to long-term career progression, especially during adolescence. Therefore, the primary purpose of the study was to evaluate Italian regional rugby union academy players’ (i.e., under-18) anthropometric and physical characteristics during a competitive season. Body mass, height, upper- and lower-body maximal strength, sprint, and high-intensity running ability were assessed in 29 elite players (backs, n = 13, forwards, n = 16). A mixed-design analysis of variance (ANOVA) for repeated measures showed that backs were shorter (ES = 0.59), lighter (ES = 0.94), stronger relative to body mass (bench press; ES = 0.60; deadlift; ES = 0.63; clean ES = 0.63; rowing ES = 0.67), and fitter (shuttle run max; ES = 0.38; shuttle run tot; ES = 0.79) than forwards. However, the forwards achieved greater sprint momentum (initial sprint momentum; ES = 0.97; maximal sprint momentum; ES = 0.98). During the season, players changed in stature, upper-body maximal strength, jumping, and high intensity running (p < 0.05), but not in body weight or lower-body maximal strength (p > 0.05). Maximal strength improved in the first part of the season, whereas jumping and sprinting performances increased in the last part of the season. Therefore, these findings highlight the importance of regularly monitoring the physical development in a long-term perspective, even suggesting that physiological adaptations are heterochronic between positional roles.

## INTRODUCTION

Rugby union is a high-intensity contact team sport that requires players to possess a diverse range of physical attributes [1]. In senior rugby union, strength, power, speed, and agility characterize players according to their position on the field. Forwards are typically the strongest, heaviest, and tallest, in order to be competitive within rucks, mauls, and lineouts [2, 3]. Indeed, even in elite junior rugby, set pieces in which forwards are involved (i.e., scrum, lineout, and kick-off) represent crucial moments for success [4]. Conversely, backs might need speed, acceleration, and agility to beat the opposition in open play [1, 3, 5]. Thus, players’ characteristic and body composition, along with technical and tactical skills, will be principally affected by the playing position [3].

Differences in strength and condition capacity (i.e., maximal strength, aerobic capacity, and repeated sprint ability) were also highlighted between junior backs and forwards. In particular, backs were faster (i.e., greater sprint velocities) and more skilled in both aerobic capacity and repeated sprint ability than forwards, in all junior categories (i.e., under 16 [U16], under 18 [U18] and under 21 [U21]) [6]. Also, changes in anthropometrics, abilities, and motor skills (i.e., high-intensity running and sprint abilities) were not equally distributed over age categories. Some of them (i.e., anthropometrics, high-intensity running ability) mainly changed among U16 and U18, whereas sprint significantly improved among U18 and U21[7], suggesting that physiological adaptations are heterochronic. In particular, variations in anthropometric and physical characteristics during a season occur at a greater rate in adolescent rugby players in comparison to senior squad members [3, 7, 8]. Factors such as full-time training, greater access to sports science knowledge, and more professional training staff have led to greater athletic development, marked increases in players’ size and body mass index, and changes in body shape over the last decades [5, 9–11].

However, physical characteristics in young rugby players may partially contribute to long-term career progression [12–14]. In fact, in contact sports (e.g., soccer, basket, rugby, and water polo), players with higher muscular strength and body dimensions are more likely to be selected at an early age [15, 16]. In particular, according to a study focused on rugby union [17], talent identification and development programmes in early age should focus on the development pathway rather than on the baseline motor skills, especially for the forwards.

In line with the Italian Rugby Federation (FIR), young players’ abilities and skills are developed in both non-residential (i.e., for U16) and residential (i.e., for U18 and U20) training centres to develop the players’ performance potential. In particular, U18 residential academies represent the most significant opportunity for entering a senior professional level in the near future. In fact, according to the international rugby union guidelines (i.e., World Rugby), around the age of 17 to 20 years, the long term player’s development approach should maximize individual preparation and performance [18]. In addition, this growth process is also stimulated by the passage from the 4 regional U18 academies to the unique U20 national academy, which can be considered as the most important talent selection for Italian rugby union players, who will definitively be assigned to elite or sub-elite categories.

For this reason, regular monitoring of physical and anthropometric changes during an entire season appears to be crucial in young rugby union players. Nevertheless, no study has monitored the anthropometric and physical characteristics in young Italian rugby union players. Therefore, the purpose of this study was to monitor anthropometric (i.e., body mass, height) and physical (i.e., upper- and lower-body maximal strength, sprint, and high-intensity running ability) characteristics in Italian regional rugby union academy players (i.e., U18) during a whole season, in relation to positional roles (i.e., backs and forwards).

## MATERIALS AND METHODS

### Subjects

Twenty-nine junior (age range = 16–18 years) rugby union players (backs, n = 13, body weight = 73.9 ± 4.8 kg, height = 176.1 ± 4.8 cm; forwards, n = 16, bodyweight = 92.3 ± 13.2 kg, height = 182.2 ± 5.1 cm) were evaluated. All players were members of the regional FIR U18 academy. During the academy development programme, players trained 5 days a week (~180 minute/ session), including gym and field sessions. Additionally, each player trained and competed with their local amateur club during the weekend. Field-based sessions included generic speed development skills, technical drills, and small-sided games, while gym-based sessions focused on general strength development, flexibility, and hypertrophy. Testing was performed after a 3-week off-season training period including full-body resistance training, aerobic conditioning running, and technical skills. Informed consent was obtained from one parent of each player participating in the study. This study was approved by the local institutional review board, and all performance data were made anonymous to ensure players’ privacy.

### Design

This longitudinal study was designed to compare junior rugby players’ characteristics between different positions (i.e., backs and forwards) across an entire competitive season (September 2016 – June 2017). Players from an Italian U18 regional academy performed a testing battery at the beginning (September 2016), in the middle (March 2017), and at the end (June 2017) of the season. Players were assessed on anthropometric (height and body mass) and physical (one repetition max [1-RM] bench press, back squat, deadlift, power clean, prone row, squat jump, armless-countermovement jump, countermovement jump, 0–10 and 0–30 m sprint and multiple shuttle test) measurements. Changes in performance between beginning (T1), middle (T2) and end of season (T3) were evaluated, to assess the longitudinal development of anthropometric and physical characteristics. All assessments were performed by the same strength and conditioning coach after 3 weeks of familiarization. To ensure a complete recovery among evaluations, players were tested at the same time, in different days over an entire week. All the exercises were regularly used in the training programmes. Before testing, a standardized 10-minute warmup including dynamic movements and stretching was completed.

### Methodology

#### Anthropometry

Body mass and height were measured to the nearest 0.1 kg and 1 cm respectively using calibrated Seca Alpha (model 813) scales and a Seca Alpha stadiometer (Seca, Birmingham, UK). Each anthropometric measurement was made in the morning and with the players wearing shorts.

#### Strength

The 1-RM bench press, back squat, deadlift, power clean, and prone row were performed to measure upper- and lower-body strength. During the 1-RM bench press, the Olympic barbell had to touch the chest, followed by a return to complete elbow extension without assistance. For the back squat, a researcher verified that players reached the thigh parallel to the floor before the execution of the concentric phase. For the deadlift, players were required to reach the complete hips and knee extension after lifting the barbell from the floor. For the power clean, players completed the repetition by catching the barbell across their shoulders with both elbows pointing forward in a standing position. For the prone row, players were in a prone position on a bench fixed to a squat rack. They completed the repetition by lifting the barbell from the bottom position (i.e., elbows extended) until it touched the bench. For all the trials, players performed 3 attempts, with a 3-minute rest between. The best 1 RM was considered for the statistical analysis. After all strength assessments, the player’s 1-RM scores were divided by the current (i.e., relative to the period of assessment) body mass to provide a more objective strength score.

#### Jumps

Squat jump (SJ), countermovement jump armless (CMJa) and countermovement jump (CMJ) were performed to assess the jump height, without and with arm swing coordination, respectively. For all exercises, players stepped into Optogait (Microgate, Bolzano, Italy) bars and held steady. Players started from a squat position for the SJ, and from standing to squatting to jumping with arms akimbo and arms swinging for CMJa and CMJ..

#### Sprint

Each player performed three 0–30 m sprints on an artificial field using a system of photocells (Witty, System, Microgate, Bolzano, Italy) with timing gates placed on 0.8 m high tripods at 0, 10 and 30 m. The players began each sprint with their front foot beside a 0.30 m cone behind the first gate. A rest time of 4–5 minutes was given between each trial. Sprint time (s) was registered for both 0 to 10 and 0 to 30 m distances. The 0 to 10 m and the 0 to 30 m splits were considered as representative of the acceleration ability and maximal velocity, respectively [19]. Velocity scores (m^-1^⋅s^-1^) were calculated for both splits by dividing the distance (i.e., 10 and 30 m) for the time taken to complete the trial. The mass of the athlete was multiplied for both 0 to 10 m and 0 to 30 m velocity scores (kg⋅m^-1^⋅s^-1^) to obtain an initial (ISM) and maximal (MSM) sprint momentum score.

#### High-intensity running

The 5 m multiple shuttle run test (MST) was used to evaluate the anaerobic capacity in team sport because the demands of the test are similar to game demands. Standard testing and warm-up protocols were applied [20] and the test was recorded. The distance covered by each subject was approximated to the nearest 2.5 m during each 30-second shuttle. Peak distance (i.e., the greatest distance covered during a 30-s shuttle) and total distance (i.e., the total distance covered during the 6 x 30-s shuttle) were assessed by a researcher and double-checked by video subsequently.

### Data Analysis

Data were presented as mean ± SDs of anthropometric and physical characteristics. Data were analysed using a 2 x 3 (position × time) mixed-design analysis of variance (ANOVA) for repeated measures to verify time (beginning, middle, and end of season), position (backs, forwards), and interaction (position and time) effects. When a significant F-value was obtained, the Bonferroni post-hoc test procedure was also performed. The sphericity assumption was tested by means of Mauchly’s sphericity test, which is applied after the Greenhouse– Geisser correction if the sphericity was primarily violated. Cohen’s d effect sizes (d) and the relative 95% CI were calculated and evaluated according to the following thresholds: < 0.2 = trivial, 0.2–0.6 = small, 0.7–1.2 = moderate, 1.3–2.0 = large, and > 2.0 = very large [21]. The level of significance was set at 5% (P < 0.05). All data analyses were performed using JASP version 0.12 software (JASP Team, http://www.jasp-stats.org/) and using the statistical package R (version 4.0.3; R Core Team, Foundation for Statistical Computing, Vienna, Austria), with the package emmeans (version1.3.2).

## RESULTS

**FIG. 1:**
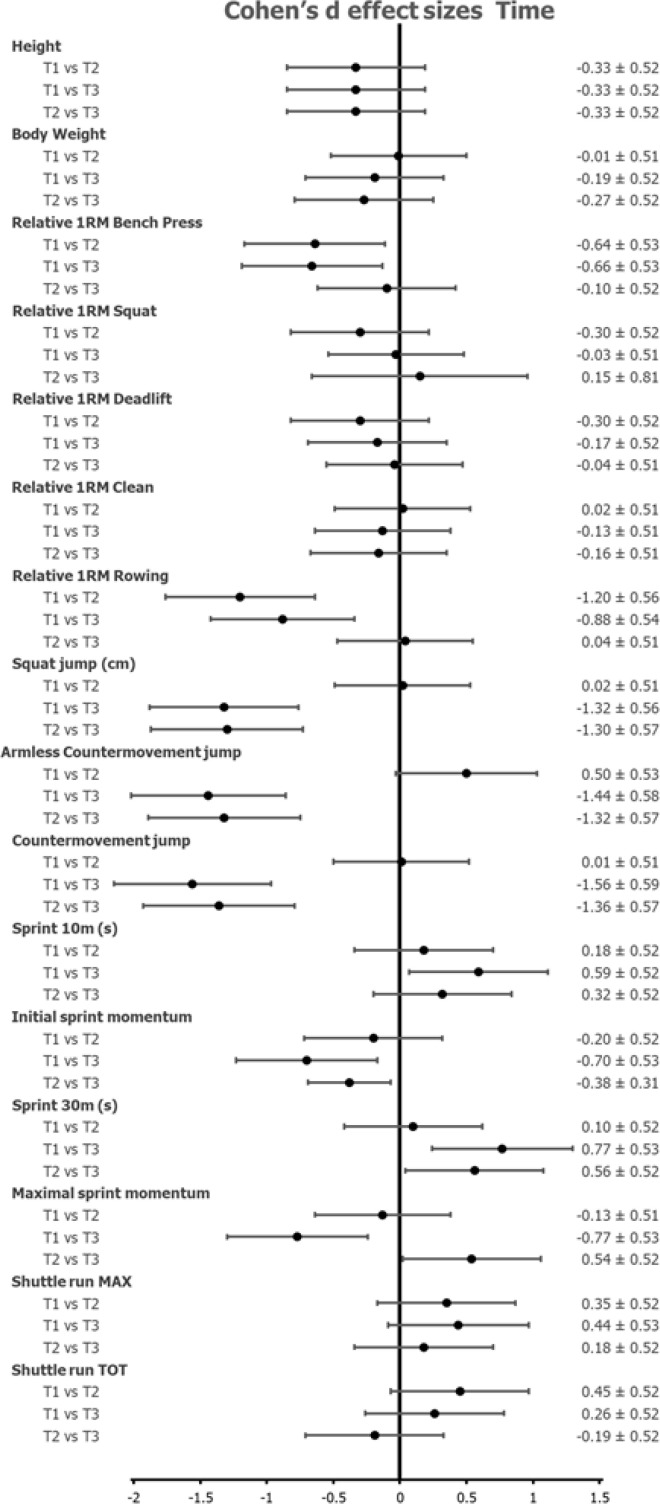
Cohen’s d effect sizes in relation to time (i.e., T1, T2, T3).

**FIG. 2:**
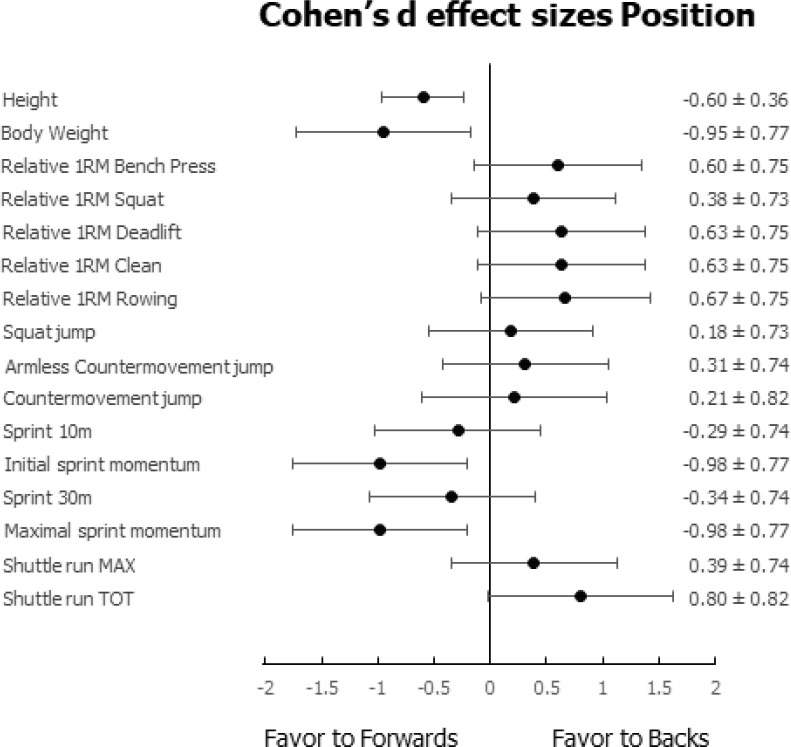
Cohen’s d effect sizes in relation to position (i.e., forwards, backs).

The mean and SD of the anthropometric and physical characteristics of back and forward players in relation to the beginning, middle, and end of the season and the relative analysis of variance outcomes are reported in Table 1, whereas the overall Cohen’s d effect sizes in relation to time (i.e., T1, T2, T3) and position (i.e., forwards, backs) are reported in Figures 1 and 2, respectively.

**Table 1:** Descriptive statistics (mean ± SD) of the anthropometric and physical characteristics at the beginning (T1)-, middle (T2)- and end-season (T3) and the relative mixed model repeated-measures analysis of variance outcomes.

	T1		T2		T3		Time effect		Position effect		Interaction effect	
	Backs	Forwards	Backs	Forwards	Backs	Forwards	F_2, 28_	p-value	F_1, 28_	p-value	F_2, 56_	p-value
Height (cm)	176.1 ± 4.8	182.2 ± 5.1	176.3 ± 5.1	182.3 ± 5.1	176.3 ± 5.1	182.3 ± 5.1	3.447	0.039	10.382	0.003	0.614	0.545
Body Weight (Kg)	73.9 ± 4.8	92.3 ± 13.2	74.1 ± 4.1	92.1 ± 12.4	74.1 ± 4.8	93.0 ± 11.6	0.845	0.417	26.032	< 0.001	0.538	0.555
Relative 1RM Bench Press (%)	107.7 ± 14.1	93.5 ± 15.5	112.7 ± 14.1	101.1 ± 10.1	115.7 ± 12.7	99.9 ± 8.9	9.907	< 0.001	10.494	0.003	0.700	0.471
Relative 1RM Squat (%)	160.9 ± 19.1	153.2 ± 33.4	164.8 ± 21.1	158.3 ± 25.9	172.1 ± 13.1	145.6 ± 8.2	0.572	0.523	4.167	0.051	3.492	0.051
Relative 1RM Deadlift (%)	179.4 ± 22.8	155.4 ± 21.3	180.8 ± 21.8	159.7 ± 17.8	181.6 ± 15.1	160.2 ± 22.1	0.613	0.485	11.511	0.002	0.106	0.819
Relative 1RM Clean (%)	96.7 ± 18.5	83.5 ± 16.1	94.4 ± 18.2	84.9 ± 11.2	104.4 ± 10.9	80.8 ± 5.8	0.949	0.376	11.769	0.002	4.993	0.017
Relative 1RM Rowing (%)	120.7 ± 14.9	105.1 ± 11.8	127.8 ± 12.8	117.2 ± 11.8	130.1 ± 11.1	114.6 ± 6.2	18.317	< 0.001	13.364	0.001	1.226	0.301
Squat jump (cm)	34.2 ± 3.9	34.6 ± 3.4	35.9 ± 3.1	33.2 ± 3.1	39.5 ± 2.8	39.2 ± 2.7	33.257	< 0.001	0.933	0.343	2.707	0.076
Armless Countermovement jump (cm)	36.3 ± 3.7	36.1 ± 3.3	37.6 ± 3.5	35.1 ± 3.09	41.1 ± 3.7	40.6 ± 1.3	37.221	< 0.001	1.214	0.280	2.260	0.114
Countermovement jump (cm)	42.1 ± 3.7	41.1 ± 3.5	42.1 ± 4.1	37.7 ± 3.65	45.9 ± 4.2	46.1 ± 2.4	44.237	< 0.001	2.745	0.109	6.202	0.004
Sprint 10m (s)	1.81 ± 0.09	1.84 ± 0.06	1.80 ± 0.05	1.82 ± 0.06	1.76 ± 0.06	1.82 ± 0.05	4.534	0.015	2.461	0.128	1.295	0.282
Initial sprint momentum (Kg⋅m^-1^⋅s^-1^)	407.1 ± 25.4	500.9 ± 63.3	409.9 ± 19.3	504.1 ± 61.8	420.1 ± 27.5	510.6 ± 58.1	6.173	0.004	27.751	< 0.001	0.186	0.830
Sprint 30m (s)	4.28 ± 0.19	4.38 ± 0.16	4.29 ± 0.11	4.35 ± 0.15	4.18 ± 0.15	4.30 ± 0.12	7.163	0.002	3.382	0.077	0.681	0.510
Maximal sprint momentum (Kg⋅m^-1^⋅s^-1^)	517.7 ± 28.0	631.4 ± 78.9	518.2 ± 25.8	635.1 ± 76.9	532.1 ± 33.3	648.4 ± 71.3	8.755	< 0.001	28.056	< 0.001	0.091	0.913
Shuttle run MAX (m)	130.6 ± 4.4	128.8 ± 4.9	130.7 ± 2.8	125.9 ± 2.0	126.5 ± 1.3	128 ± 1.8	5.291	0.017	4.301	0.048	8.562	0.002
Shuttle run TOT (m)	746.4 ± 17.8	720.9 ± 30.8	742.2 ± 15.8	705 ± 16.4	730.6 ± 17.3	721.2 ± 12.6	3.405	0.056	18.225	< 0.001	5.943	0.010

*Notes:* T1, beginning-season; T2, middle-season; T3, end-season.

### Anthropometry

A repeated measure ANOVA reported differences between subjects for height and body weight. In particular, backs were shorter and lighter than forwards throughout the entire season. However, no difference between subjects emerged in post-hoc comparisons. No time × position interaction effect was found.

### Strength

Backs were stronger than forwards in bench press, deadlifting, cleaning, and rowing. Changes in bench press and rowing were observed during the season. In particular, differences emerged between T1-T2 and T1-T3 for both bench press and rowing.

### Jumps

Repeated measure ANOVA revealed changes in jump tests during the season. In particular, differences emerged between T1-T2 and T2-T3 for SJ and CMJa, and between T2-T3 as well for CMJ.

### Sprint

Repeated measure ANOVA showed no difference between positions for 0–10 m and 0–30 m sprint. Changes in 0–10 m sprint and 0–30 m sprint were observed during the season. In particular, differences were found between T1-T3 for 0–10 m and 0–30 m sprint, and between T2-T3 as well for 0–30 m sprint. Differences were found between T1-T3 for initial (i.e., 0–10 m) and maximal (i.e., 0–30 m) sprint momentum, and between T2-T3 as well for maximal sprint momentum. Between position, backs achieved less initial and maximal sprint momentum compared to forwards.

### High-intensity running

Repeated measure ANOVA revealed that backs achieved higher peak and total distance in the shuttle run test.

## DISCUSSION

This is the first study reporting the reference data on the anthropometric and fitness profiles of youth Italian rugby union players. In particular, results were provided for the under 18 FIR academy, which can be considered as the most crucial phase before competing in senior elite teams. As the main findings, this study showed that the under 18 players changed in upper-body pushing (i.e., relative 1 RM bench press), upper-body pulling (i.e., relative 1-RM rowing), jumping (i.e., SJ and CMJ), sprinting (i.e., 0–10 and 0–30 m), and high intensity running (i.e., shuttle run), but not in body weight and lower-body pushing (i.e., 1 relative RM squat, deadlift and power clean) over the training season. Although stature showed a statistically significant change (p = 0.039), this was deemed non-significant by Cohen’s post-hoc testing.

Since high level competition in rugby union requires players to have greater lean mass and lower percent body fat [22], we can hypothesize that only the ratio between the fat mass (in decreasing) and lean mass (in increasing) changed, even though no changes in body weight occurred in this sample. In line with this speculation, we could assume that the improvements in upper-body relative strength (1-RM weight/body mass) were due to the increased lean mass. However, the hypothesized lean mass changes along the youth rugby union categories (e.g., from U16 to U20) are not clear [7, 23], and further investigations, including assessment of body composition, are required. On the other hand, differences in stature and body weight seem to be influenced by position. In fact, according to previous studies [24–26], backs were shorter and lighter than forwards, in line with the senior rugby players [1, 3, 5] and the worldwide selection process since a young age [25].

Upper-body relative maximal strength, differently from jumps and sprints, improved constantly over the season, for both pushing and pulling skills. In particular, the magnitude of increase was greater over the first period of the season (i.e., from T1 to T2) than the second one (i.e., from T2 to T3). This may be due to the adaptation to the resistance training completed in the first period of the season [27, 28]. Because of the greater adaptation potential, the young players, with less resistance training experience, could benefit more from the resistance training in the first part of the season [29]. In particular, players starting the regional FIR U18 academy began to stress the upper body more than they did for the lower body resistance training compared to the previous training programmes in their clubs of origin. Therefore, we can speculate that the improvements that occurred in upper body relative strength are due to both higher training volume and greater adaptation potential.

Although no difference in jump height was observed between backs and forwards overall, improvements occurred over the season. Different trends were observed for SJ and CMJa compared to CMJ. In particular, improvements in jump height emerged at the end of the season compared to the middle and the beginning, while no difference was observed between the beginning and the middle season. This trend was already observed in different age groups (i.e., from under 13 to under 19) [30], showing an increase in performance with age. Biological maturation [7] and expertise [31] could explain the observation, because improvements emerged only at the end of the season. Moreover, the trend for CMJ was even the opposite in the middle of the season, especially for the forwards. In fact, the forwards decreased their jump height between the beginning and the middle of the season, despite it increased between the middle and the end of the season. Despite the fact that these data are controversial with respect to those of the SJ and CMJa, it can be hypothesized that the inclusion of an arm swing while performing a countermovement jump is able to elicit different results [31–33]. In fact, an arm swing generates an additive lower-extremity independent effect in the CMJ, increasing the jump height [33], while the CMJa isolates lower-extremity force production. Therefore, the CMJ may mostly provide pertinent information about long-term changes in sport-specific performance, whereas the CMJa may be principally oriented at detecting acute changes in neuromuscular fatigue and the athlete’s readiness [31].

Similar to the jump height, small to moderate differences were observed in sprinting between the beginning and the end of the season. In contrast with previous research [6, 34], backs were not different from forwards in sprinting velocity, although the former were lighter but with higher relative strength. In fact, in line with the literature [35, 36], greater relative lower body strength led to better sprint and jump performances. Body mass should be taken into account when considering sprinting in rugby because of the need to enhance dominant collisions during the sport specific performance [37, 38]. Consequently, backs had less sprint momentum than forwards. Nevertheless, it is not possible to provide an evident cause-effect interpretation for this result, and thus we can only speculate that backs’ sprinting skills were not so good with respect to those of heavier forwards participating in the present study. In fact, maximum transfer from resistance training to sprint performance also requires a specific exercise programme [39], including plyometric training and traditional sprint-training drills. Moreover, backs were also too light (~18 kg) to counterbalance sprint momentum differences, although they were relatively strong in 1-RM strength testing.

High-intensity running ability assessed by the 5-m multiple shuttle test was greater in the backs in comparison to the forwards. Backs demonstrated greater performance for the total distance covered within the 6 x 30-s shuttles, as well as for the peak distance covered during a single 30-s shuttle. Comparison between backs and forwards highlight two different development pathways over the season. According to the moderate differences in body mass between backs and forwards (d = 0.95), higher body mass probably impacts negatively on the forwards’ ability in performing intermittent shuttle running. As a consequence, academy coaches might focus on selecting heavier forwards to increase collision forces during impacts in rugby competition, being aware of the damaging trade-off for high intensity running performance.

This study presents longitudinal data for positional differences in anthropometric, strength, jumps, sprint, and high intensity running ability for U18 regional academy rugby union players. Therefore, relatively to this category of players, the findings demonstrate that: i) upper-body relative strength and jump abilities can change over the season; ii) height, body mass, upper- and lower-body relative strength and high intensity running ability can differ between backs and forwards; iii) backs in this sample were not faster than forwards even if they were lighter and relatively stronger; and iv) backs’ sprint momentum was lower than that of forwards. Nevertheless, further research is required to analyse training load over time, to be more aware whether an optimal training periodization to enhance sprint performance can be provided. In addition, future research should evaluate effective interventions aimed at increasing sprint velocity, especially for backs. Finally, to develop an ecological understanding of adolescent players’ physical development, a comprehensive testing battery, including anthropometrics, body composition, strength, power, and locomotor specific skills (i.e., sprint and high intensity running) should be promoted, to better understand the similarities and variations observed in athletes competing in different disciplines [40]. Due to the heterogeneous trends that occur during the physiological adaptation in adolescents’ physical abilities, coaches, managers, and stakeholders in general should be aware of the importance of regularly monitoring the physical development in a longterm prospective study. Moreover, both coaches and strength and conditioning trainers should individualize training programmes to stimulate players’ development for both technical/tactical and physical perspectives.

## CONCLUSIONS

These findings suggest that physical characteristics develop at different rates over a season in Italian regional academy rugby union players. Players also experienced different seasonal improvements in relation to the tactical role. To ensure the best individual development, coaches, as well as strength and conditioning trainers, could emphasize athletic skills (i.e., sprinting, jumping) alongside maximal strength development within an appropriate periodization. Practically, players should perform strength and power (e.g., back and front squats, jump squats, power clean, split jerk, glute ham rises), plyometric (e.g., broad jump, multiple broad jumps, drop jumps, maximal hopping), and sprint-specific (e.g., sled and uphill sprints) workouts for improving physical performance. According to the findings in this study, strength improvements could occur more in the middle of the season compared to the plyometric and the sprint-specific performance, which are expected to occur more at the end of the season. Moreover, from a development perspective, coaches should be aware that backs are shorter, lighter, but stronger and faster than forwards. Therefore, individualized training programmes should be provided to the different positional roles to maximize players’ physical development, which can be associated with the tactical skills of backs and forwards [41], as well as with those of an entire team, such as strong defence, tackling, scrumming, breaking the defensive line, and high occurrence of possessions during the attacking phase [42].
